# Protein Kinase CK2α’, More than a Backup of CK2α

**DOI:** 10.3390/cells12242834

**Published:** 2023-12-14

**Authors:** Mathias Montenarh, Claudia Götz

**Affiliations:** Medical Biochemistry and Molecular Biology, Saarland University, Building 44, 66421 Homburg, Germany; claudia.goetz@uks.eu

**Keywords:** protein kinase CK2, phosphorylation, subcellular localization, protein–protein interaction, review, CK2α isoforms

## Abstract

The serine/threonine protein kinase CK2 is implicated in the regulation of fundamental processes in eukaryotic cells. CK2 consists of two catalytic α or α’ isoforms and two regulatory CK2β subunits. These three proteins exist in a free form, bound to other cellular proteins, as tetrameric holoenzymes composed of CK2α_2_/β_2_, CK2αα’/β_2_, or CK2α’_2_/β_2_ as well as in higher molecular forms of the tetramers. The catalytic domains of CK2α and CK2α’ share a 90% identity. As CK2α contains a unique C-terminal sequence. Both proteins function as protein kinases. These properties raised the question of whether both isoforms are just backups of each other or whether they are regulated differently and may then function in an isoform-specific manner. The present review provides observations that the regulation of both CK2α isoforms is partly different concerning the subcellular localization, post-translational modifications, and aggregation. Up to now, there are only a few isoform-specific cellular binding partners. The expression of both CK2α isoforms seems to vary in different cell lines, in tissues, in the cell cycle, and with differentiation. There are different reports about the expression and the functions of the CK2α isoforms in tumor cells and tissues. In many cases, a cell-type-specific expression and function is known, which raises the question about cell-specific regulators of both isoforms. Another future challenge is the identification or design of CK2α’-specific inhibitors.

## 1. Introduction

CK2 (formerly known as casein kinase 2) is a highly conserved serine/threonine protein kinase, which is ubiquitously expressed in eukaryotic organisms. It is an important enzyme among the more than 500 protein kinases encoded in the human genome [1,2] as it is implicated in the regulation of fundamental processes within a eukaryotic cell. The number of proteins that are phosphorylated by CK2 is increasing rapidly [3,4]. In general, protein kinases are regulated by phosphorylation or dephosphorylation, by signal molecules and their second messengers, and by reversible association with regulatory subunits. CK2α and CK2α’ are also active in the absence of CK2β. There are diverse functions of CK2β, such as providing stability to CK2α isoforms and regulation of substrate-specific and complex formation to generate tetramers consisting of CK2α_2_/CK2β_2_, CK2αα’/CK2β, or CK2α’_2_/CK2β_2_ complexes. There is a very tight binding of CK2α or CK2α’ to CK2β. Moreover, in contrast to many other protein kinases, CK2 is not a member of a vertical signal transduction cascade. It acts more in a lateral manner, influencing factors of several signaling cascades. The enzyme is reported to be constitutively active and not directly regulated by growth factors, hormones, and cellular signaling molecules [5,6]. There is, however, increasing evidence that CK2 is regulated by phosphorylation, acetylation, and O-linked glycosylation; by aggregation into hetero-oligomers consisting of two CK2α or CK2α’ and two CK2β subunits and the formation of oligomeric complexes of this tetramer; and by variable subcellular localization and complex formation with cellular or viral proteins [7,8,9,10,11,12,13]. CK2 seems to be implicated in the regulation of fundamental biological processes, such as the regulation of proliferation, survival, differentiation, and the regulation of cellular metabolic pathways (for reviews see: [14,15,16,17,18,19,20,21,22]). Accordingly, it is not surprising that CK2 plays a role in many human diseases, such as diabetes [15,23,24], obesity [25,26,27], and human malignancies, such as cancer or viral infections [10,22,28,29,30,31,32,33,34,35,36,37]. The implication of CK2 in human diseases has stimulated the search for potent and specific inhibitors of the protein kinase activity of CK2 [38,39,40,41]. Some of these inhibitors reached clinical trials (for review see [39,42]). Although there is a great number of reports describing substrates of CK2, its influence on cellular signaling pathways, and even on viral infections, there are many open gaps in understanding how CK2 is regulated and how this enzyme can fulfill its multiple functions. One key to understanding these multiple functions may be the presence of two catalytic subunits, CK2α and CK2α’. The vast majority of reports are based on studies on CK2α or do not discriminate between both CK2 isoforms. The aim of the present review is the look at CK2α’-specific structures and functions.

## 2. Genes and Proteins

The human genome contains four CK2 loci, three active genes, and one pseudogene [43]. The active CK2α locus (CSNK2A1) was found on chromosome 20p13, the CK2α’ locus (CSNK2A2) on chromosome 16p21, and the CK2β locus (CSNK2B) on chromosome 6p21 [43,44]. The analysis of the promoter regions of CK2α and CK2β revealed common binding sites for transcription factors, which raised the question about a common regulation of the expression. Indeed, the group of Pyerin and Ackermann found transcriptional coordination of the expression of CK2α and CK2β [45] by regulation of the phosphorylation of the transcription factors Sp1 and Ets1 by the CK2 holoenzyme but not CK2α. A later study revealed that neither CK2α nor CK2α’ bind to the promoter regions of the CK2α and CK2β genes [46]. However, inhibition of the kinase activity of CK2 is implicated in the regulation of its own expression supporting the original model by Pyerin and Ackermann.

**Figure 1:**
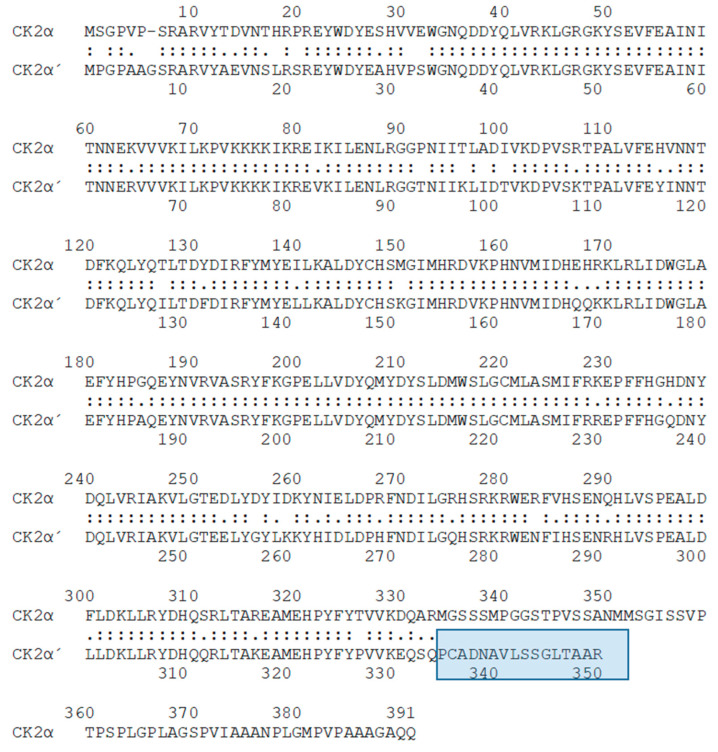
Alignment of the human CK2α and CK2α’ subunit (https://www.ebi.ac.uk/Tools/psa/lalign, accessed on 5 December 2023). The unique C-terminal region of CK2α’ is highlighted in blue.

Amino acid sequence analysis has shown that CK2α and CK2α’ share a conserved catalytic domain and a general conservation in vertebrates with some differences in the C-terminal regions [47,48] (Figure 1). Knock outs of CK2α, and also of CK2β, in mice are lethal at early embryological stages [49,50]. In contrast to these results, CK2α’ knock-out mice are viable. However, male CK2α’ knock-out mice showed globozoospermia with an altered shaping of the nuclear heads of spermatozoa leading to round-headed infertile spermatozoa [51]. Furthermore, CK2α and CK2β seem to be essential for proliferation and embryonal development whereas CK2α’ seems to be dispensable for these features. These results support the idea about the specific functions of CK2α and CK2α’ and that the two catalytic subunits cannot functionally substitute each other.

There is some indication that the individual subunits are free of the other subunits [52]. The dominant form of CK2, however, consists of two catalytic α and/or α’ subunits, which are complexed with two non-catalytic β-subunits to build α_2_β_2_, αα’β_2_, or α’_2_β_2_ heterotetramers [53]. Beside the heterotetramer with a molecular weight of around 150 kilodaltons, several multimers of the heterotetramer are reported [54,55]. Moreover, all three subunits can bind to other cellular or viral proteins [9,56,57,58]. Human CK2α consists of 391 amino acids (Figure 1). On SDS polyacrylamide gels, CK2α runs at a molecular weight of around 42 kDa [59]. The human CK2α’ subunit consists of 350 amino acids. On SDS polyacrylamide gels, the CK2α’ protein runs at a molecular weight of 39 kDa. One has to be aware that CK2α can also be present at a molecular weight of 38 kDa due to C-terminal proteolysis [60]. Missing subunit-specific antibodies, as well as the instability of CK2α’, have for a long time impeded the analysis of CK2α’ specificity. A breakthrough was the generation of CK2α’-specific antibodies in 1999 by using the CK2α’-specific peptide (EQSQPCADNAVLSSGLTAAR) as an antigen [61].

Olsen et al. succeeded in the expression of a stable and soluble maltose binding protein (MBP) tagged CK2α’, which was incubated with CK2β to form a CK2α’_2_β_2_ holoenzyme. The MBP tag was then cleaved within the holoenzyme. The resulting holoenzyme was active as a protein kinase [62]. This CK2α’_2_β_2_ holoenzyme showed nearly the same Km value for the synthetic substrate peptide as the CK2α_2_β_2_ holoenzyme. Both holoenzymes could use ATP, as well as GTP, as phosphate donors. However, both holoenzymes showed a striking difference in thermostability. After 2 min at 45 °C, the CK2α-containing holoenzyme did not lose enzymatic activity whereas the CK2α’-containing holoenzyme had lost about 50% of its activity [62]. Another striking difference was the autophosphorylation of CK2β at Ser2 and Ser3 [63] and the autophosphorylation of CK2α in the presence of polybasic compounds [64] in the CK2α-containing holoenzyme compared to the CK2α’-containing holoenzyme. Gel filtration experiments showed that the affinity between CK2α’ and CK2β is significantly lower than the affinity between CK2α and CK2β [65]. Furthermore, gel filtration experiments showed that the polybasic compound spermine leads to a dissociation of the oligomeric CK2 holoenzyme [62]. Although these are mainly in vitro experiments, proteins with polybasic stretches might regulate CK2α autophosphorylations in different subcellular compartments. One of these proteins might be p53, which was shown to bind to CK2β [66]. The CK2β binding region on the polypeptide chain of p53 is basic and may, therefore, influence CK2 kinase activity similar to spermine.

The vast majority of CK2 exists in tetrameric complexes [67]. Velocity sedimentation analysis and electron microscopy of CK2 from *Drosophila melanogaster* revealed the presence of high molecular weight forms of CK2 at physiological ionic strength. Filamentous forms of CK2 appeared at high salt concentrations [68]. These early observations already indicated that the polymerization of CK2 might play a role in the regulation of the enzyme. The Battistutta group constructed holoenzymes composed of two in vitro expressed CK2β subunits and two in vitro expressed C-terminal truncated versions of CK2α, which account for a molecular mass of 130 kDa. Structure analysis showed the existence of trimeric and high molecular aggregates depending on the ionic strength in the solution [54,69]. The polyamine spermine destabilized the oligomeric structure, probably by a competition with CK2β for binding to the basic stretch of CK2α [54,62]. This observation is comparable with the activation of CK2 kinase activity by polyamines [70,71]. Oligomerization of the holoenzyme is necessary for an autophosphorylation of CK2β [8]. Interestingly, CK2β isolated from cells is extensively autophosphorylated [72], indicating the presence of considerable amounts of the oligomeric form of the CK2 holoenzyme. There was no CK2β autophosphorylation when CK2α’, instead of CK2α, was used for the formation of the holoenzyme. This observation goes along with an absence of CK2 aggregates of the CK2α’β_2_- holoenzyme [65]. At a physiological ion concentration, there might be an equilibrium between inactive oligomeric forms of CK2 and the monomeric CK2α_2_β_2_- holoenzyme. These results might explain functional differences between CK2α and CK2α’ holoenzymes. Oligomerization of the holoenzyme seems to be a regulatory mechanism because this might limit the access to substrates due to steric hindrance. On the other hand, by in vitro experiments with different forms of CK2 and six different CK2 kinase inhibitors, it was shown that the CK2 holoenzyme was more sensitive towards the inhibitors than the free catalytic subunits [65].

Originally, CK2α’ was preferentially found in mouse brains and testes [52]. A study by Ceglia et al. described a predominant expression of CK2α in the mouse brain by a factor of eight over CK2α’ [73]. The expression of the CK2α’ protein was significantly higher in the hippocampus and the prefrontal cortex than in other brain regions. Alvarado-Diaz et al. have analyzed the expression of CK2α’ during rat spermatogenesis. By Western Blot analysis, they found that CK2α’ and CK2β are expressed in testes from birth to adulthood. Immunohistochemical analysis shows that CK2α’ is located in the nucleus of Sertoli cells from young animals; whereas, it is found in the cytoplasm in older animals [74]. Interestingly, mature epididymal spermatozoa express CK2α’ in the acrosome and CK2β in the flagellum. Thus, these results support the idea of a cell-type-specific expression of CK2α’, a dynamic localization within the cells, and the presence of CK2α’ in the absence of CK2β.

Rebholz et al. generated floxed CK2α, CK2α’, and CK2α/α’ mice where Cre was expressed in the postnatal forebrain under the control of the CaMKIIα promoter. Floxed Cre-CK2α/α’ mice died around birth. The floxed Cre-CK2α’ mice were viable with no obvious biochemical or behavioral phenotype [75]. In the Drd1a-Cre CK2α KO medium spiny neurons, there was a small increase in the expression of CK2α’, indicating a compensatory function in the absence of CK2α.

To further exploit the role of individual CK2 subunits for cell survival, the Pinna group used CRIPR/Cas9 technology. They succeeded in generating C2C12 myoblasts, which were originally published as having lost kinase activity [76]. Later on, an N-terminally deleted form of kinase active CK2α’ was detected in the CK2 knock-out C2C12 myoblasts cells [77]. Interestingly, the knock out of CK2α and the expression of the N-terminally truncated CK2α’ resulted, also, in a reduced level of the non-catalytic CK2β subunit. Knocking out the CK2β subunit increased CK2α expression significantly; whereas, CK2α’ expression was reduced. These alterations were also detected at the mRNA level, indicating that CK2β regulates the expression of the catalytic CK2α subunits on the transcriptional level, which supports early observations by Pyerin and Ackermann [43]. These results might further indicate that the CK2α_2_/β_2_ holoenzyme is implicated in the regulation of the expression of CK2α’. Later on, it was reported that the kinase active truncated CK2α’ binds to CK2β. This mutant form of CK2α’ showed reduced thermostability as compared to wild-type CK2α’ [77]. The N-terminally deleted form of CK2α’ had a limited number of substrates compared to full-length CK2α’. A phosphoproteome analysis of the CK2α^(−/−)^/ΔCK2α’ C2C12 myoblasts [77] surprisingly showed only a slightly altered CK2-specific phosphoproteome [78] compared to wild-type cells. Treatment of these cells with two different CK2 inhibitors, CX-4945 or GO289, led to a significant reduction in cell viability. The phosphoproteome generated by the KO cells was still sensitive to CK2-specific inhibitors. These results indicated that the residual CK2 kinase activity generated by the N-terminally deleted form of CK2α’ is sufficient to support cell viability [77].

## 3. Interacting Partners of CK2α’

It is well known that both CK2α and CK2α’ strongly interact with CK2β in yeast two-hybrid assays [79,80]. CK2α’, however, binds CK2β more than 10 times weaker in vitro than does CK2α [59,81].

Varjosalo et al. cloned the tagged cDNAs for thirty-two human kinases, including the cDNA coding for CK2α’, and transfected these constructs in HEK293 cells to generate single cells expressing one of the tagged kinases. In a two-step purification procedure, proteins bound to the kinases were further analyzed by LC-MS. In total, 62 proteins were identified as binding partners of CK2α’. These proteins were, however, not cross-checked against binding to CK2α. Thus, it remains unclear whether they bind exclusively to CK2α’ or to both catalytic CK2 subunits [82]. There are cellular proteins that bind to CK2α but not to CK2α’. One of these proteins is PP2A, which in vitro binds to free CK2α but not to the holoenzyme. Moreover, CK2β prevents the binding of PP2A to CK2α [83]. The same results were obtained by co-immunoprecipitation experiments with overexpressed CK2α. The binding site on the polypeptide chain of CK2α to PP2A was identified as a sequence that is not identical to the corresponding amino acid sequence in the polypeptide chain of CK2α’. Therefore, it was not surprising that CK2α’ binding to PP2A was not described [84].

Another protein binding to CK2α, but not to CK2α’, is CKIP-1. By yeast two-hybrid experiments, in vitro binding experiments, and co-immunoprecipitation experiments, Bosc et al. identified CK2 interacting protein 1 (CKIP-1) as a binding partner of CK2α but not of CK2α’ [85]. Yeast two-hybrid screens with different regions of CK2α revealed that the carboxy-terminal CK2α missing amino acids 332–391 is not sufficient for the binding of CKIP-1 to CK2α. Immunoprecipitates of CKIP-1 contain a CK2 protein kinase activity that is lower than in CK2 immunoprecipitates. This result might indicate low amounts of the CK2 associated with CKIP-1 or a kinase activity that is inhibited by CKIP-1. At least in vitro, CKIP-1 does not alter the activity of CK2 and, therefore, one might conclude that only minor amounts of CK2α bind to CKIP-1.

In a yeast two-hybrid screen, the motor neuron protein KIF5C was found as a binding partner of CK2 [86]. This interaction was confirmed by co-immunoprecipitation and co-sedimentation analysis using cell extracts from human neuroblastoma cells. Immunofluorescence analysis showed that KIF5C was mainly localized in the cytoplasm where, also, CK2α’ and CK2β were found; whereas, CK2α was mainly found in the nucleus. Pull-down experiments with GST-tagged KIF5C revealed that CK2α’ bound to KIF5C but not CK2α [86]. KIF5C inhibited CK2α’ but not CK2α kinase activity.

In non-small-cell lung cancer, by a co-immunoprecipitation experiment, breast cancer metastasis suppressor 1 (BRMS1) was identified as a CK2α’ but not a CK2α binding partner [87].

Table 1 shows a summary of the unique binding partners of CK2α or CK2α’, respectively.

## 4. Biological Functions of CK2α’

In the early days of CK2 research, different subcellular localizations for the CK2 subunits were reported [88,89,90,91,92,93]. In times when no CK2α’ specific antibodies were available due to overlapping sequences of CK2α and CK2α’, there was one antibody directed against the unique C-terminus of CK2α [94]. All other CK2 antibodies recognized both proteins, CK2α and CK2α’. CK2α and CK2β were mainly localized to the cytoplasm; whereas, the antibody recognizing both catalytic subunits stained both the nucleus and the cytoplasm in asynchronously growing cells. These results may suggest that the nuclear CK2α’ is mainly free of CK2β. Yu et al. further found that CK2α’ is mainly nuclear in the G_1_ phase of the cell cycle and cytoplasmic in the S phase [94]. Belenguer et al. [95] and Filhol et al. [92] also reported different subcellular localization depending on the cell cycle. From these studies, it was suggested that CK2α’ might play a role in DNA replication, transcription, and nuclear and nucleolar re-organization; whereas, CK2α might mainly play a role in the regulation of the organization of the cytoskeleton [94].

Having already shown that CK2α isoforms are found at different places within the cell cycle and during the life cycle of a cell and that they interact with different cellular proteins, possible specific functions of both CK2α isoforms remain to be discussed.

**Figure 2:**
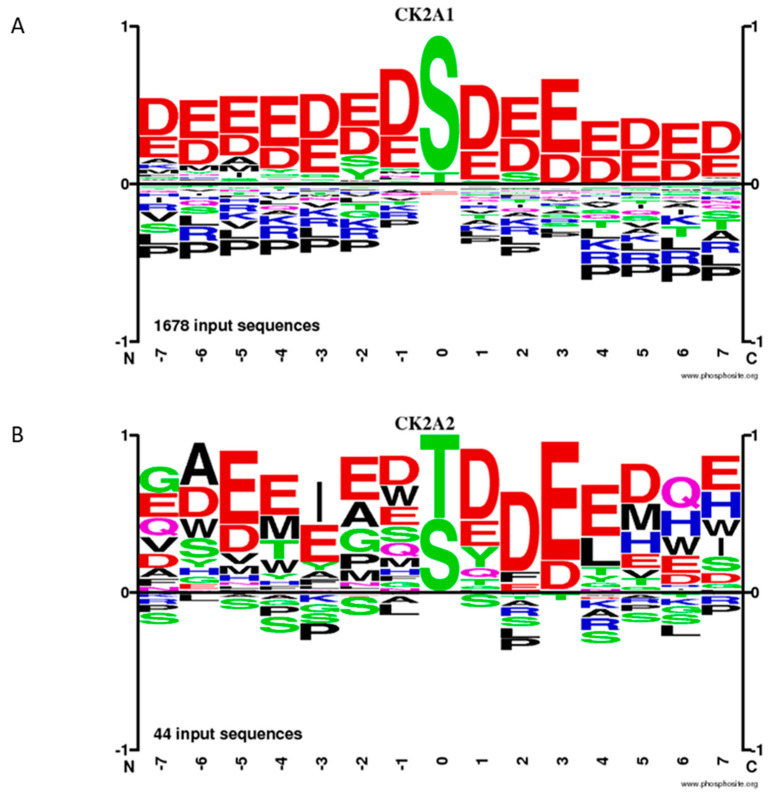
Frequency of phosphorylation sites of CK2α (**A**) and CK2α’ (**B**), according to the database phosphosite.

It is an interesting observation that there are obviously differences in the consensus sequences for the CK2α and CK2α’ phosphorylation sites, respectively. Based on 1678 input sequences, CK2α prefers the amino acid serine within an acidic environment; whereas, CK2α’ phosphorylates serine and threonine residues nearly as well. Moreover, the sequences upstream of threonine or serine are less acidic in the case of CK2α’ compared to CK2α ([96] and https://phosphosite.org, visited 1 December 2023) (Figure 2). Most of the phosphorylation sites were identified by in vitro phosphorylation experiments. Under in vivo phosphorylation conditions, the CK2 phosphorylation sites might differ because of other kinases that phosphorylate overlapping sequences.

CK2α’ exhibits a striking preference over CK2α for caspase-3 phosphorylation in cells. This preference is not observed with recombinant proteins in vitro. CK2β negatively regulates caspase-3 phosphorylation in cells. Caspase-3 is phosphorylated by recombinant CK2 at T174 and T176 [97], which protects procaspase-3 from cleavage by caspase-8 and caspase-9. This protection seems to be a mechanism by which CK2 could protect cells from apoptosis [97]. Later on, the same group reported that CK2α’ preferentially phosphorylated caspase-3 [98]. With chimeras of CK2α/CK2α’, it was shown that only CK2α-HA constructs containing amino acids 45–300 of CK2α’ phosphorylated caspase-3 greater than CK2α-HA. The unique C-terminal domain does not play a role in dictating specificity towards caspase-3 [98]. Although it is known that CK2α’ binds CK2β more than 10 times weaker than CK2α does, CK2β blocks the phosphorylation of caspase-3.

One striking feature is the specific expression of CK2α’ during the differentiation of cells and in some human diseases. CK2α’ is preferentially expressed in the late stages of the spermatogenesis of mice. Male mice with a knock out of CK2α’ are infertile with oligospermia and globozoospermia. The primary defect in CK2α’ knock-out testes is a specific abnormality of the anterior head shaping of elongating spermatids [51]. Recently, it was shown that FSIP2, which is associated with the development of the acrosome and flagellum in humans, seems to be involved in the expression of CK2α’ [99]. Spermatozoa from patients carrying FSIP2 mutations showed a down-regulation of the expression of CK2α’ [99]. Mutations in FSIP2 contribute to globozoospermia and, thus, the early observations by the Seldin group have been extended. Furthermore, the testes of CK2α’ mice have increased numbers of apoptotic cells. It might be an interesting question whether the absence of CK2α’ in sperm can be used as a marker of infertility.

DNA-dependent protein kinase (DNA-PKc) is an enzyme necessary for non-homologous end-joining during the repair of DNA double-strand breaks. By analyzing two different glioblastoma cell lines, M059J, which lacks DNA-PKc, and M059K, which expresses DNA-PKc [100,101], Olsen et al. demonstrated an increase in CK2α’ in DNA-PKc-deficient M059J cells, both at the mRNA and at the protein level [102]. No such increase was observed for CK2α. The mechanism of this increase remains, however, an enigma.

In the last couple of years, CRISPR/Cas9 technology has helped to detect more specific functions of the CK2 subunits. Salizzato et al. have analyzed the influence of the deletion of individual CK2 subunits on myogenic differentiation [103]. CK2α KO C2C12 myoblast cells exhibit a substantial down-regulation of CK2β, as also shown by [104]; whereas, CK2α’ KO cells showed very similar levels of CK2α and CK2β to the control cells. The differentiation program of CK2α’ KO cells is similar to the control cells. However, the differentiated CK2α’ KO cells are mainly mono-nucleated [103]. CK2α’ seems to be essential for the plasma membrane localization of caveolin-3 and myomixer, indicating an influence of CK2α’ on membrane fusion. Thus, these results showed another specific function of CK2α’, which is different from CK2α functions.

Lettieri et al. succeeded in generating immortalized mouse neurons where CK2α, CK2α’, or CK2β had been knocked down [105]. The knock down of the catalytic CK2 subunits induced a decrease in CK2β levels. This observation is not unexpected and supports early observations about a rapid degradation of CK2β in the absence of the catalytic CK2 subunits [76]. CK2β KO cells showed a significant decrease in CK2α’ expression, which supported the idea that CK2β is responsible for the stabilization of CK2α’. Alternatively, or in addition, the CK2α_2_/β_2_ holoenzyme might play a role in the regulation of the transcription of CK2α’. All three KO cells showed a decrease in the proliferation rates. The CK2β KO cells showed a more pronounced decrease, suggesting that the corresponding holoenzymes seem to play a major role in cell proliferation. Furthermore, there seems to be a strong influence of CK2α’ and CK2β on cell migration and of CK2α’ on cell adhesion, as demonstrated in the corresponding KO cells [105].

Huntington’s disease (HD) is a neurodegenerative disease caused by the modification of the Huntingtin-2 gene [106]. Elevated CK2 protein kinase activity was found in mutant Huntingtin-expressing cells [107]. CK2α’ mRNA and protein levels are induced in HD mouse models and in cells of patients with Huntington’s disease [108]. Genetic depletion or pharmacological inhibition of CK2α leads to a decreased Huntingtin aggregation, restored mitochondrial gene expression, and improved motor behavior and lifespan [108,109]. In heterozygous mice lacking one allele of CK2α’, Yu et al. showed that alpha-synuclein is a substrate for CK2α’ and that, in particular, Ser129 phosphorylation of alpha-synuclein was increased in medium spiny neurons (MSNs) and increased the striatal synapse density in HD mice [109]. Altogether, these results showed a positive effect of a reduction in CK2α’ on neuroinflammation and motor behavior.

Another CK2α’ specific function was reported by Kishihara et al. in mouse immature cardiomyocytes [110]. The authors showed that angiotensin II activated Cav1.2 channels. This activation was inhibited by the CK2 inhibitor quinalizarin, indicating a contribution of CK2 to the activation of the Cav1.2 channels. The knock down of CK2α’ or CK2β, but not of CK2α, suppressed the angiotensin II activation. Interestingly, the authors also found an increase in the level of CK2α’ during the maturation of the heart. Co-immunoprecipitation experiments showed the binding of CK2α’ and CK2β to Cav1.2. By knock-down experiments, p27 was identified as an inhibitor of CK2α’ and, thereby, the activation of the Cav1.2 channels. Phosphorylation of p27 at Tyr88 leads to the abrogation of the inhibitory effect of p27 on CK2α’_2_/β_2_. These results are in agreement with the results obtained by Hauck et al. [111]. Activation of Cav1.2 is finally achieved by its CK2α’_2_/β_2_ phosphorylation at Thr1704 [110].

Treatment of mouse cells with serum resulted in elevated mRNA expression for CK2α’ with a peak at 4 h after treatment. The expression kinetics correlate with an elevated kinase activity, as measured with the common substrate peptide (RRRADDDSDDDDD). The ectopic expression of CK2α’ together with activated H-ras resulted in the transformation of rat primary embryo fibroblasts. CK2α’/H-ras transformed cells show a faster growth rate than cells transformed with H-ras alone. Interestingly, the mRNA expression of CK2β increased with the same kinetic as CK2α’. The expression of the CK2α mRNA showed a less pronounced increase at 2 h after the serum treatment of the cells than the mRNA for CK2α’ [112].

Later on, more and more reports appeared showing the role of CK2α’ in cancer cells. Using the ONCOMINE database [113], Ortega et al. analyzed the expression of the CK2 genes in six cancers with a high mortality rate in the U.S.A. [114]. They found an overexpression of CK2α but not of CK2α’ in small-cell lung carcinoma cells. Invasive and non-invasive breast cancer cells showed an under-representation of CK2α’ compared to normal tissue. While high levels of CK2α correlated with lower overall survival rates, there was no significant influence of CK2α’ on survival in all breast cancer cells. Also, in ovarian cancer, CK2α’ was down-regulated. In prostate cancer, CK2α was up-regulated in all three subtypes; whereas, CK2α’ was up-regulated only in one prostate cancer subtype. In summary, a deregulated expression of CK2 subunit expression seems to be an important factor during tumorigenesis and, in particular, for prognosis. There seems to be, however, no general up- or down-regulation of CK2α’ and the other subunits. Up- or down-regulation varies between subtypes of the different cancer types [114].

There is an overexpression of CK2α’ in non-small-cell lung cancer (NSCLC) compared with adjacent non-cancerous tissue [87]. Phosphorylation of breast cancer metastasis suppressor 1 (BRMS1) by CK2α’ was promoted by TNF. Moreover, co-immunoprecipitation experiments revealed that the BRMS1 protein binds to CK2α’ but not to CK2α. The TNF-induced CK2α’ phosphorylation of BRMS1 resulted in its nuclear export and ubiquitin-dependent degradation in the cytoplasm. Lowering the level of BRMS1 led to increased metastasis and poor clinical prognosis [87].

An elevated expression rate of CK2α’ and protein expression has also been described for hepatocellular carcinoma cells (HCCs). The higher expression is significantly associated with tumor size, tumor stage and tumor differentiation, and a lower survival rate [115]. Down-regulation of CK2α’ in HCC cell lines by infection with lentivirus expressing shCK2α’ led to elevated apoptosis and repressed cell migration. Overexpression of CK2α’ resulted in an activation of the NF-κB pathway in HCCs [115]. A comparison with CK2α is missing

Another functional difference between CK2α and CK2α’ has been reported in the prostate tumor cell line LNCaP [116]. The prostate-restricted and androgen-regulated gene NKX3.1 has been linked to prostate cancer. Recombinant NKX3.1 is known to be phosphorylated in vitro by CK2 on T89 and T93. Blocking the kinase activity of CK2 by apigenin or DRB resulted in a decrease in NKX3.1 stability. The knock down of CK2α’ but not CK2α also led to a decrease in the NKX3.1 steady-state level. In an in-gel kinase assay, CK2α’ phosphorylated recombinant human and mouse NKX3.1 [116]. Furthermore, the siRNA knock down of CK2α’ diminished NKX3.1 accumulation in LNCaP cells. Anion exchange chromatography revealed the existence of free CK2α’ in addition to CK2α’ in the complex to CK2β.

By knock-out studies of CK2α and CK2α’ in the human neuroblastoma cells SK-N-BE and in the human osteosarcoma cells U2OS, the group of Maria Ruzzene analyzed the individual knock out on metabolic and cellular functions. The expression of CK2β was unchanged in CK2α’ knock-out cells while it was reduced in CK2α knock-out cells [104]. The clonogenic potential of CK2α’ knock-out U2OS clones was reduced compared to wild-type cells. In general, the proliferation rates and survival of CK2α and CK2α’ cells were reduced compared to wild-type cells. Wound healing was reduced for CK2α’ and especially CK2α KO cells compared to wild-type cells; although, there are differences between SK-N-BE and U2OS cells. Extra-cellular lactate levels were reduced in CK2α knock-out cells and weakly reduced, if at all, in CK2α’ knock-out cells. There was a shift to anaerobic glycolysis in CK2α knock-out cells [104].

The knock down of CK2α’ by a lentivirus expressing siRNA reduces the viability of Huh7 and MHCC97H cells. In a nude mice model, Huh7 and MHCC97H shCSNK2A2 cells, tumor volume, and weights are lower. Furthermore, overexpression of CK2α’ activates the NFκB pathway [115].

**Figure 3:**
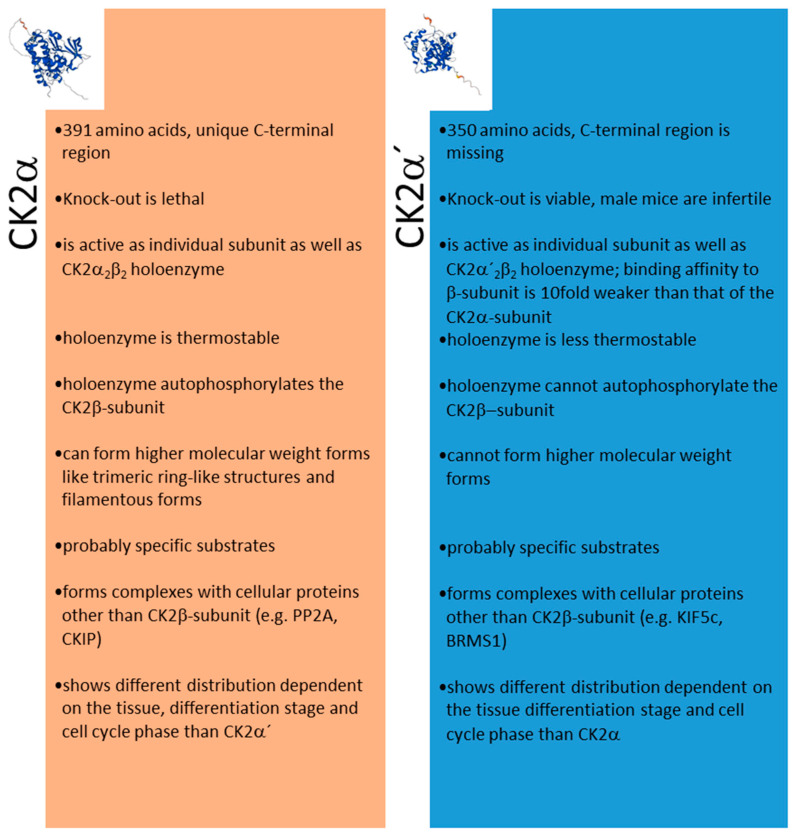
Different features of CK2α and CK2α’. Structures in the insert are from the AlphaFold Protein Structure Database (ebi.ac.uk, acessed on 27 October 2023).

A summary of the different features of CK2α and CK2α’ is shown in Figure 3.

## 5. Inhibitors of CK2α’

Due to the observations about individual features and specific functions of CK2α compared to CK2α’, it was not surprising that several groups attempted to characterize CK2α’-specific inhibitors. Using in vitro expressed CK2α or CK2α’, Baier et al. identified compounds that inhibited CK2α’ but not CK2α, such as tricin or scutellarin [117]. Due to the missing crystal structures of these compounds with CK2α subunits, the underlying mechanism for this specificity has not been elucidated. Using an oxidative-resistant CK2α’ mutant for a crystalline study, the bivalent CK2-inhibitor KN2 was designed, which had a high affinity for both catalytic CK2 subunits [118]. The group of Joachim Jose established an auto-display assay using proteins displayed on the cell surface of *E. coli* [119]. This assay was successfully used for the development of new enzyme assays [120] and also for the expression of CK2α, CK2β, and the tetrameric holoenzyme of CK2 [121]. In addition, this assay was used for the expression of CK2α, CK2α’, or CK2α’/CK2β on the *E. coli* cell surface in order to identify CK2α isoform-specific inhibitors [122]. After testing 13 different known CK2 inhibitors, so far, no preference for one of the isoform-specific forms CK2α/CK2β or CK2α’/CK2β could be detected. However, this approach seems to be promising for finding new CK2 isoform-specific inhibitors.

Another approach to identifying CK2 isoform-specific inhibitors might be crystallographic structure analysis of CK2 isoforms together with kinase inhibitors. Crystal structure analyses of CK2α’ are rare due to the poor solubility and the formation of the thin needle-shaped crystals [123]. So far, there seem to be structural differences in the CK2α–CK2β interface compared to the CK2α’–CK2β interface, which might allow the design of CK2α’-specific inhibitors [124]. Recently, a mutant CK2α’ was used for crystallographic studies with different well-known inhibitors. Werner et al. found subtle differences for the reduced binding of the CK2 inhibitors SGC-CK2-1 to CK2α’, which correlates with the reduced IC_50_ value of SGC-CK2-1 for CK2α’ inhibition compared to CK2α [41,125].

## 6. Conclusions

The present review shows that CK2α’ is not simply a backup for the CK2α isoform. There is obviously a reciprocal regulation of the expression of the different CK2 subunits, not directly but indirectly via the phosphorylation of implicated transcription factors. In addition, differential affinity for CK2β and complex formation with CK2β lead to an influence on the stability of the CK2α isoforms. The catalytic domains of CK2α and CK2α’ show 90% homology. CK2α also has a C-terminal sequence, which is missing in the CK2α’ protein. So far, however, no clear statements have been made about the functions of this unique C-terminal sequence. Other than differences in the phosphorylation and acetylation of CK2α and CK2α’, different higher molecular forms of the CK2 holoenzyme are formed, which leads to different stability; phosphorylation of CK2 subunits; and, due to the more complex structure, different access to substrates, as well as to CK2 kinase inhibitors.

Depending on the cell type, different subcellular localizations of CK2α isoforms, as well as the respective holoenzymes with CK2β, are reported. Overall, the subcellular localization of CK2 subunits exhibits a certain dynamic during the cell cycle or during the developmental stages.

In particular, CK2α’ appears to interact differently with cell-cycle-regulating proteins. Across the board, there appear to be differential interactions with cellular proteins. The results obtained with model cells of human diseases, as well as with cell material from patients, including tumors, indicate different functions of CK2α and CK2α’.

Different molecular biology methods to knock down or to knock out one of the two CK2α isoforms have recently revealed very interesting isoform-specific functions. Especially, the results in human diseases, including cancers, increase the pressure to develop CK2α isoform-specific inhibitors. Initial approaches and results are promising indications for future use in the treatment of human disease. 

## Figures and Tables

**Table 1:** Binding partners of CK2α or CK2α’.

	CK2β	Tubulin	PP2A	CKIP-1	KIF5C	BRMS1
CK2α	+	+	+	+	-	-
CK2α’	+	+	-	-	+	+
